# Facilitating Advance Care Planning Conversations Among Patients With Cancer and Their Care Partners Utilizing a Conversation Game: A Pilot Study

**DOI:** 10.1002/cnr2.70250

**Published:** 2025-06-17

**Authors:** Kylee Kimbel, Michael Hayes, Morgan Bucher, William A. Calo, Tullika Garg, Monika Joshi, Hannah Kuntz, Terrence E. Murphy, Erika VanDyke, Emily Wasserman, Lauren J. Van Scoy

**Affiliations:** ^1^ Penn State College of Medicine Hershey Pennsylvania USA; ^2^ Penn State Cancer Institute Hershey Pennsylvania USA; ^3^ Geisinger Medical Center Danville Pennsylvania USA

**Keywords:** advance care planning, advance directives, cancer, health games, medical decision‐making, qualitative research

## Abstract

**Background:**

Patients with cancer should engage in decision‐making throughout the course of their illness and treatment. Current guidelines recommend early, frequent advance care planning (ACP) conversations among clinicians, patients, and care partners (CPs) and advance directive (AD) completion. However, only 55% of patients with cancer have completed such directives, suggesting the need for interventions to increase rates of ACP. The *Hello* game has been shown to be effective in promoting ACP in several populations but has not been tested in patients with cancer or their CP.

**Aims:**

To assess the acceptability of *Hello* and determine the modifications necessary for use in cancer populations.

**Methods and Results:**

This convergent mixed methods study involved seven dyads (7 patients with cancer and their 7 CPs to total *n* = 14); dyads played *Hello* in groups of 2–4. Post‐game, dyads completed satisfaction and acceptability questionnaires and focus groups. Qualitative thematic analysis was performed; quantitative data was summarized.

Patients' mean age was 56.4 years—43% were female, 57% had genitourinary cancer, and 43% had breast cancer. Three themes emerged from both patient and CP focus groups (*n* = 14 individuals): (1) Participants enjoyed the group dynamics and relating to peers when playing *Hello*; (2) *Hello* serves as a helpful conversation starter; (3) modifications could help tailor *Hello* for use in cancer context—particularly adding more questions about quality of life and mental health. The patient focus groups had an additional theme: (4) Patients with localized cancer may have a different experience with *Hello* when compared to those with advanced cancer.

**Conclusion:**

*Hello* was well‐received by dyads, and their feedback was used to tailor *Hello* for patients with cancer and their CPs. Next steps for this project include assessing the acceptability of the modified game.

**Trial Registration:** clinicaltrials.gov: NCT06028152

## 1 Introduction

Patients with cancer often face difficult medical decisions over the course of treatment. These decisions include whether to pursue aggressive treatments that may involve side effects that could affect quality of life or whether a comfort‐focused, palliative approach is preferred [1]. It is critical that patients with cancer and their care partners consider and address preferences for cancer‐focused treatment along with their overall medical care, documenting their preferences in advance directives (legal documents that name surrogate decision‐makers [SDM] and describe medical preferences) [2] in an ongoing process called advance care planning (ACP) [3]. ACP is an ongoing process that involves individuals considering their values, goals, and beliefs related to their medical care, having discussions about their wishes with one's family and/or healthcare team, and then completing advance directives [3].

In the absence of ACP, patients with cancer who are unable to communicate their preferences risk losing autonomy over these decisions and, as a result, are less likely to receive goal‐concordant care and more likely to receive undesired, costly, or overly aggressive care, particularly at the end of life (EOL) [4, 5]. Additionally, care partners can experience enduring anxiety, depression, and post‐traumatic stress when called upon to make decisions on behalf of their loved one as an SDM [6, 7, 8]. Guidelines from the American Society of Clinical Oncology call for ACP to be performed at the time of diagnosis and throughout the cancer continuum [9]. Despite these recommendations, many patients in active treatment for cancer have not engaged with ACP, with one study finding only 55% of patients in active treatment for an advanced cancer have completed an advance directive or had an ACP conversation [10]. While exact data on the uptake of ACP in patients with cancer is limited, reasons for not engaging in ACP have been well studied. They include the inherent discomfort patients and care partners face when considering EOL issues, lack of opportunity to have conversations about ACP, and gaps in knowledge about how to engage in ACP [11, 12]. Interventions that address these barriers are needed to increase rates of ACP in patients with cancer. One evidence‐based intervention that could address this barrier is *Hello*, a conversation game that involves 32 open‐ended questions related to ACP that invites participants to have ACP discussions in small groups [13].*Hello* has been studied in multiple populations, including patients with chronic illness and their care partners and among Black, Hispanic, and South Asian populations, but has yet to be evaluated with patients with cancer [13, 14, 15, 16, 17, 18].

In prior studies, participants have found the game provides a safe and effective forum for meaningful conversations about EOL issues and has inspired the sharing of rich perspectives that were highly valued by participants [13, 17, 19, 20]. It has also been shown to motivate high rates of subsequent ACP behaviors [15, 17, 18]. In a recent national study, of 220 Black participants, 98% of the Black participants completed at least 1 ACP behavior (e.g., discussing EOL issues with loved ones or clinicians, or review of ACP resources) after playing *Hello*; 67% completed at least 3 ACP behaviors, and 48% completed, reviewed, or revised an advance directive [20]. No participants reported any adverse events, excessive burden, or negative emotional effects from the game experience [13, 14, 17, 18, 21].

The goal of this convergent mixed methods study was to: (1) explore the feasibility and acceptability of using the *Hello* game with patients with cancer and their care partners; (2) learn what types and stages of cancer the game would be the most impactful; and (3) use findings to inform modifications made to *Hello* to address cancer‐specific ACP needs.

## 2 Methods

### 2.1 Study Design

This convergent mixed methods study was conducted at the Penn State Cancer Institute in Hershey, PA and was approved by the Penn State University Institutional Review Board (STUDY00023029). The study recruited seven dyads consisting of patients with cancer and their chosen care partners (total sample of *n* = 14) between October 31st, 2023 and January 31st, 2024. Care partners were chosen by the patient and defined as any person they would want to be involved in their medical decisions. Dyads attended one study visit that involved completing questionnaires about their readiness to engage in ACP, playing the conversational game *Hello*, completing questionnaires about satisfaction and acceptability of the game, and participating in a focus group about perceptions of the game.

### 2.2 Recruitment and Inclusion Criteria

Convenience sampling was used to recruit dyads. Patients were identified by reviewing clinic schedules of partnering providers. Patients aged 18 years and older were eligible if they met the following inclusion criteria: (1) diagnosed with breast, lung, or genitourinary cancer of any stage; (2) either in active treatment for their cancer diagnosis or have received treatment in the past year at the Penn State Cancer Institute; and (3) had self‐reported ability to read, write, and speak in English. Since the objective of this study was to explore the types and stages of cancer for whom the intervention is most relevant, we opted to include a broad array of diagnoses and stages.

Potential participants were confirmed eligible by their oncology provider and then contacted by research assistants by phone, patient portal message, or in‐person at clinic visits. Interested patients provided written informed consent and were asked to identify a care partner to participate in the study, defined as someone they would want to be involved in making their medical decisions should they be unable to do so themselves. Eligibility criteria for care partners were an age of 18 years old or older and self‐reported ability to read, write, and speak in English.

### 2.3 Measures and Intervention

Four game events were held with 7 dyads (to total *n* = 14 individuals). Event scheduling was completed after the dyads were enrolled and was determined by the mutual availability of the enrolled dyads and the research team. At the study visit, participants completed pre‐intervention measures that included demographics and baseline ACP engagement (via the validated, 4‐item ACP Engagement [ACPE‐4] survey) [22, 23]. The ACPE‐4 yields an average score from 4 items rated on a five‐point Likert scale (1 = lowest engagement; 5 = highest engagement) assessing readiness to perform each of the following behaviors: (1) legally name a surrogate decision‐maker, (2) discuss preferences for EOL care with the surrogate decision‐maker, (3) discuss preferences for EOL care with the healthcare team, and (4) legally document wishes for medical care at EOL. In addition to the ACPE‐4, care partners completed the validated 17‐item ACP engagement survey for surrogate decision‐makers (ACPE‐17‐SDM) that was previously adapted from the ACP engagement survey to be relevant for SDMs (but has yet to be validated as a 4‐item version) [24]. The ACPE‐17‐SDM score is calculated as the average of 17 items asked on a 5‐point Likert scale (1 = lowest engagement; 5 = highest engagement) assessing the care partner's knowledge, self‐efficacy, contemplation, and readiness for serving as a SDM [24].

After completing pre‐game questionnaires, *Hello* game rules were explained by the research assistant and participants played for 45 min. The *Hello* game consists of 32 open‐ended questions that prompts conversation about values for EOL care and ACP [13, 14, 15]. An example question is: “Which is more frightening to imagine: Suffering the worst physical pain of your life or not getting a chance to say goodbye to your family?” Players take turns reading each question aloud and then silently answering the questions before sharing and discussing their response with others. While sharing is not required, it is encouraged and allows for connections between participants. As answers are shared, conversation ensues, and the group discusses their responses in an open‐ended manner. During the game, players may exchange “thank you” chips with other players as a token of appreciation for an answer shared or to acknowledge a poignant or even humorous comment. Prior to game play, a coin is flipped and hidden until the end of the game when it is then revealed whether the player with the most chips (heads) or least chips (tails) is the ‘winner’.

After the game, participants completed questionnaires assessing their satisfaction with the conversation (8 items asking whether the participants found the conversation satisfying, productive, useful, etc.) [25, 26] and the acceptability of the conversation (4 items, see Table 2). The conversation satisfaction score is the average of 8 items, whereas each of the acceptability items is evaluated independently and does not yield an overall score. Finally, patients and care partners participated in separate focus groups at the end of each event. Focus groups were led by trained research assistants to explore experiences playing *Hello* and whether modifications were needed to tailor the game for the cancer context. Focus groups (*n* = 4 groups inclusive of 7 patients; *n* = 4 groups inclusive of 7 care partners to total *n* = 14) were audio‐recorded and transcribed verbatim by a professional transcription service. At the completion of the study visit, all participants were provided with an ACP workbook that includes an AD, instructed on how to complete it, and received a $25 stipend for participation.

### 2.4 Analysis

Due to the qualitative nature of this study design, the sample size was determined based on the estimated number of participants needed to reach data saturation (i.e., the point when no new information is gleaned from additional qualitative data collection). Based on prior studies, we anticipated data saturation to occur after three focus groups and thus recruited four focus groups for each group (patients, care partners) to help ensure saturation [13, 17].

Descriptive summary statistics for patients' and care partners' demographic characteristics and survey questionnaire measures are reported using frequency counts with percentages for categorical measures and means with standard deviations for continuous measures. SAS software Version 9.4 (SAS Institute Inc., Cary, NC, USA) was used to summarize the quantitative data measures.

Patient and care partner focus groups were analyzed separately using the same process. First, two analysts (KK and MB) independently reviewed the four focus group transcripts to confirm data saturation. We confirmed that data saturation was reached after review of 50% of the data (*n* = 2 transcripts). Next, an initial codebook was created that included categories, codes, and definitions. This preliminary codebook was used to independently code 50% of the data (*n* = 2 transcripts) using MAXQDA 2024 [27]. To determine inter‐rater reliability, Cohen's kappa was calculated. Coding discrepancies were resolved by study team discussion to reach a final Cohen's kappa of 0.82 for patient focus groups and 0.76 for care partner focus groups. Then the remaining data was coded. Coding reports were reviewed, and preliminary themes were created. These preliminary themes were reviewed and edited by a licensed psychologist who specializes in caring for patients with cancer (MH) and the senior qualitative researcher and ACP content expert (LJV). The group convened to discuss final themes and select exemplar quotations.

## 3 Results

### 3.1 Participant Characteristics

**FIGURE 1:**
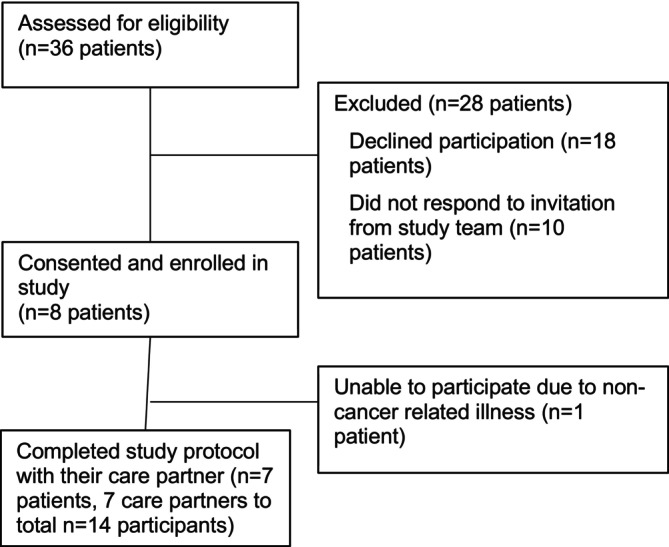
Patient participant flow diagram.

Of the 36 patients found eligible during screening, 18 declined to participate, 10 did not respond to the invitation to participate, 8 consented, and 7 patients consented and selected a care partner, resulting in 7 dyads (*n* = 14 participants; Figure 1).

The most common reasons for patients declining participation were lack of interest (5/18; 28%) and concerns about time burden (4/18; 22%). Other reasons included not wanting to talk about ACP and EOL issues, living at a distance from the medical center where the study visit took place, and progression of disease that precluded participation.

Characteristics of the 14 participants are shown in Table 1. The mean age of the patients with cancer was 56 years (SD 16.3). Most were male (57%), identified as white (86%), and had genitourinary cancer (57%). The mean age of care partner participants (all of whom were spouses) was about 56 years (SD 15); 57% were female, and 57% identified as white.

**TABLE 1:** Demographic characteristics of patient (*n* = 7) and care partner (*n* = 7) participants.

Characteristic	Patient (*n* = 7)	Care partner (*n* = 7)
Gender, *n* (%)		
Male	4 (57.1)	3 (42.9)
Female	3 (42.9)	4 (57.1)
Non‐binary or neither of the above	0	0
Age in years, mean (SD)	56.4 (16.3)	55.9 (15.1)
Cancer's primary site of origin, *n* (%)		
Breast	3 (42.9)	N/A
Genitourinary	4 (57.1)	N/A
Cancer disease stage, *n* (%)		
Stage 1	1 (14.3)	N/A
Stage 2	2 (28.6)	N/A
Stage 3	1 (14.3)	N/A
Stage 4	3 (42.9)	N/A
Race, *n* (%)		
American Indian/Alaskan Native	0	0
Asian	0	1 (14.3)
Black/African American	1 (14.3)	1 (14.3)
Native Hawaiian or Pacific Islander	0	0
White	6 (85.7)	4 (57.1)
Other, multiple races indicated	0	1 (14.3)
Ethnicity, *n* (%)		
Not hispanic/latino	7 (100)	7 (100)
Hispanic/latino	0	0
Rural/urban, *n* (%)		
Urban	6 (85.7)	6 (85.7)
Rural	1 (14.3)	1 (14.3)
Highest level of education, *n* (%)		
Did not finish high school	0	0
High school diploma or G.E.D.	3 (42.9)	0
Trade, tech, or vocational credentials	1 (14.3)	0
Some college	0	2 (28.6)
Associate's degree	0	2 (28.6)
Bachelor's degree	1 (14.3)	1 (14.3)
Graduate or postgraduate degree	2 (28.6)	2 (28.6)
Marital Status, *n* (%)		
Married, engaged, common law, long‐term partnership	7 (100)	7 (100)
Single (never married)	0	0
How would you describe your general health overall?, *n* (%)		
Excellent	1 (14.3)	2 (28.6)
Very good	2 (28.6)	4 (57.1)
Fair	4 (57.1)	1 (14.3)
Poor	0	0
Very poor	0	0
Overall quality of life, *n* (%)		
1–Life is distressing	0	0
2	0	0
3	0	0
4–Life is so‐so	1 (14.3)	0
5	2 (28.6)	2 (28.6)
6	2 (28.6)	4 (57.1)
7–Life is great	2 (28.6)	1 (14.3)
Is your care partner named as your decision‐maker in a legal document?, *n* (%)		
Yes	4 (57.1)	N/A
No	3 (42.9)	N/A

### 3.2 Quantitative Questionnaire Data

Table 2 demonstrates that patients and care partners reported similar levels of pre‐intervention readiness to engage in ACP behavior for themselves, with mean scores of 3.46 (SD = 1.11) and 3.43 (SD = 1.46), respectively. Care partners reported moderate levels of readiness to engage in ACP behavior as an SDM for their loved one with cancer, with a score of 3.41 (SD = 1.26).

**TABLE 2:** Scores of pre‐ and post‐ intervention measures.

Measure	Scoring	Mean patient score (SD)	Mean care partner score (SD)
*Pre‐intervention measure*			
ACP Engagement survey 4 item	1 = I have not thought about it; 5 = I have already done it	3.46 (1.11)	3.43 (1.46)
ACP Engagement Survey 17 item	1 = lowest engagement; 5 = highest	N/A	3.41 (1.26)
*Post‐intervention measure*			
Conversation acceptability measures			
I found the conversation activity to be an acceptable way to have an end‐of‐life conversation.	1 = strongly disagree; 7 = strongly agree	5.57 (2.07)	5.86 (0.69)
I found the conversation activity to be burdensome.	1 = strongly agree; 7 = strongly disagree	6.43 (0.53)	6.00 (1.00)
How likely are you to recommend this conversation activity to a friend or family member?	1 = not likely at all; 10 = extremely likely to recommend)	7.71 (1.98)	7.29 (1.98)
How likely are you to recommend this conversation activity to an individual with cancer?	1 = not likely at all; 10 = extremely likely to recommend)	8.71 (1.60)	8.14 (1.68)
Conversation Satisfaction	1 = lowest satisfaction; 7 = highest satisfaction	6.36 (0.60)	6.07 (0.55)

Both patients and care partners reported high satisfaction with *Hello*, as shown by mean conversational satisfaction scores of 6.36 (SD = 0.60) and 6.07 (SD = 0.55) for patients and care partners, respectively (Table 2).

### 3.3 Qualitative Focus Groups Themes

Four themes emerged from the patient focus groups and three themes emerged from the care partner focus groups. Three themes were overlapping.

#### 3.3.1 Overlapping Themes From Patient and Care Partner Focus Groups

##### 3.3.1.1 Theme 1

**Both patients and care partners equally enjoyed the group dynamics of Hello and the opportunity it gives to relate to others with shared experiences and to learn from their collective perspectives**. Some participants thought it was challenging to start these conversations with strangers, but ultimately felt that the presence of another person with cancer and their care partner helped to facilitate these discussions.

Yeah, I think it was helpful with a group. It's different dynamics, not just one‐on‐one. That maybe makes it difficult sometimes for some people, but for today's [discussion], it was really good kind of dynamics that we can kind of really talk openly (Care partner).

Another noted how it was helpful to have individuals with whom they could relate and who could relate to them on serious issues, such as mortality.

Yeah, I felt relaxed, because I was in a room with people who didn't have to explain that we might die in a little while (Patient).

Another patient said,

I think it's easier to talk about cancer with people that understand your feelings, your desires (Patient).

Similarly, some care partners found value in the group dynamic:

I liked [the game] because I felt we had kindred spirits here that we were maybe, I don't know, on the same page a lot. Ours [cancer journey] has been going on longer, but [their cancer journey] has been more traumatic maybe (Care partner).

To facilitate these connections more effectively, a few participants suggested allocating dedicated time for introductions and establishing norms before starting the game:

But it might have been a little bit more comfortable upfront had we just had an ice breaker or some time to say, what's your background or whatever that opening conversation may have been. That came with time so I would say maybe it would be good if there was time allotted for that upfront (Patient).

Specifically, to be mindful of how different individuals might react to the conversation and topics, participants wanted to know more about the conditions and cancer stage of the other players in their event group.

Well, I wouldn't want to feel like I wanted to celebrate where I am if the person across from me was not celebrating. I would feel more awkward or uneasy and I would feel bad if I said something that, not intentionally, would hurt somebody. So, I think either there would have to be kind of like a known stage, where everybody knows where everybody is because I wouldn't want to say something that would hurt somebody (Patient).

#### 3.3.2 Theme 2

**Hello may be a helpful conversation starter with a good combination of different types of questions**. Some participants appreciated the game format and noted that having a game to facilitate these conversations made approaching the topics less burdensome or uncomfortable.

It gave a good starting point for a lot of conversation about all kinds of things. So, I think that starting point makes a difference for something. It's hard to get into those conversations while just sitting and watching TV in the evening (Patient).

Similarly, another participant said,

*But I think the [*Hello*] book is [going to] have a lot of good guidelines, because it'll take that awkwardness away to say that I wanted to ask you something about the book (Care partner)*.

Someone else noted that the form of the game itself made it less intimidating as a conversation starter,

…it's like a little booklet, so it doesn't look really serious. It's kind of approachable (Care partner).

A few participants commented on the variety of questions and appreciated how the game was designed with some questions being more serious and some more lighthearted and thus easier to answer than others (i.e., emotional pacing).

The questions we went through, I think, were a good mix. They were a mix of some fun questions and some more serious questions. Some like I said earlier, just kind of digging a little deeper into what the next steps would be or what the end and some that you could make really lighthearted (Patient).

Another participant said,

I liked the way that it was set up in the way that it wasn't just melancholy question after melancholic sadness. I liked that there was like, what would your last meal be? And that kind of thing sprinkled it and I did enjoy it. And I think that was a nice way to not make it so sad to think about (Care partner).

Others noted that some topics had come up in discussions between them in other contexts, which was seen favorably.

We have talked about maybe half of the questions and also that was good for us to start thinking about (Patient).

Others noted that some of the topics were new to them.

I thought the questions were more guided to stuff we normally don't think about or talk about as far as end of life and all the– there was a lot of questions concerning that. What do I want from my epitaph? What do I want with my body? The stuff I normally don't think about. So that part was interesting (Care partner).

#### 3.3.3 Theme 3

**Modifications can help tailor Hello for use by patients with cancer**. Although participants had primarily positive comments about the game (Themes 1, 2), they also identified opportunities to better tailor the game for persons facing cancer. For example, some participants felt that mental health topics could be better highlighted for patients with cancer.

Maybe [including] the mental health aspect of it. Because sometimes when you're going through treatment, you're just so busy going through treatment, but when the treatment is over and it kind of all hits you, how do you deal, things like that, if it's more cancer‐related (Patient).

A different participant said,

Maybe addressing dark thoughts, if that makes sense because of pumping unnatural hormones into the body. That's a huge side effect (Care partner).

Some participants noted that patients with cancer have a need for different conversations about care as it relates to their diagnosis and care plans. For example, one participant suggested a question specifically about the role of hospice in end‐of‐life care.

And maybe it's later in the [game] book but along those lines if that's the goal, what are your thoughts on or how do you determine or we discussed hospice care, things like that? (Patient)

One participant also brought up the idea of having differing question booklets for people with cancer and their care partners.

I think you mentioned earlier that having one [version] for caretakers and one for both actually and one for a person with cancer might be helpful, because some of the questions I felt, not many, just maybe a couple of questions along the way, I didn't feel apply to me, because my experience was so different than theirs (Care partner).

Finally, participants also provided feedback about the planned title for the tailored version of *Hello* (*Hello, Cancer*), but participants did not favor this title, as expressed by this participant,

*When I think of Hello Cancer, I don't think of fun or having fun. I'm kind of mixed on it, but I don't like the word cancer (Patient)*.

Another patient agreed,

Because I don't like to talk or see that C word. Yeah, I think Hello is more inviting than Hello Cancer. Nobody wants to say hello to cancer (Patient).

#### 3.3.4 Theme Unique to Patient Focus Groups

##### 3.3.4.1 Theme 4

**Different treatment goals—curative vs. palliative—likely shape the conversations, decisions, and ultimately the experiences of patients playing *Hello***. As such, patients noted that these unique features across the cancer continuum may warrant differing versions of the game for differing stages of cancer.

I mean, if you say adapt [the game], I think, adapt at different stages of cancer, yes… Maybe if you have stage four, maybe focus in more of a fun and lightening kind of way, [rather] than morbid questions that they're faced with already and probably answered (Patient).

Participants also mentioned the importance of recognizing how conversations about ACP can be more emotional for those with advanced cancer.

It might be emotional. You give a person a question that does have three months to live, and physically they have three months to live, that's going to probably impact them really hard. So, I'd have different questions for different staging (Patient).

#### 3.3.5 Finding Unique to Care Partner Focus Group

While it did not appear commonly enough to rise to the level of a theme, in one focus group, participants noted that the impact of cancer goes beyond the patients to affect care partners, and that care partner issues should be addressed in *Hello*. Participants mentioned specific relationship changes that come along with a spouse undergoing treatment for cancer (e.g., reduced physical intimacy).

The other dark question that comes in here is, how does the touching, the feeling, the hugs, the kisses, all of that stuff that you do with your partner that just doesn't exist anymore. It just doesn't exist. How do you feel about that? How do you unpack those things? That is not easy. I struggle with it every single day, getting mad at her because she can't feel what I feel but yet her brain is not there (Care partner).

## 4 *Hello* Game Modifications

Our findings supported using the *Hello* game to engage patients with cancer in ACP and to help them navigate sensitive topics around medical decision‐making. However, based on qualitative feedback from the participants, the current version of the *Hello* game requires several important modifications to make the game more applicable to patients with cancer and their care partners. These modifications centered around including the care partner's experience during a loved one's cancer diagnosis, addressing overt fears associated with a cancer diagnosis, and incorporating more game questions related to palliative care and quality of life issues.

For example, in response to participant suggestions to include more content related to palliative care, we collaborated with the game designer (Common Practice LLC) to add a new question: “If a healthcare provider asked you to define ‘quality of life,’ what recent day would you tell them about?”

Another participant recommendation was to include a question that directly addressed the mental health challenges and the toll that a cancer diagnosis places on patients and their care partners. To address this recommendation, we added a question to help participants consider and express anger (“When was the last time you got angry?”), as one way to open conversation about the mental health aspects of a cancer diagnosis.

There were several comments from patients with cancer and their care partners to remove one *Hello* question that asked what they would want their last meal to be as they found this question particularly jarring. While other did like the question (see Theme 2), we opted to remove the question because several found it to invoke thoughts of ‘prisoners’. The fully revised version of the *Hello* game for use in future studies with patients with cancer and their care partners is shown in Appendix 1.

## 5 Discussion

In this exploratory study, we found that the *Hello* game was well‐received, acceptable, and appropriate for use with patients with cancer and their care partners. Our findings corroborate prior work in non‐cancer populations demonstrating *Hello* stimulates sensitive conversations in a safe forum [13, 14, 15, 17, 18]. Considering the importance of ACP conversations between patients with cancer and their care partners, these findings are encouraging as the game may offer an effective way to engage patients in ACP conversations to empower and motivate them to complete ACP behaviors (including ADs), as has been the case in other study populations [20].

*Hello's* efficacy may be attributed, in part, to its unique gamified approach to ACP engagement. Games have been well studied as an approach to discuss serious health topics [28, 29], such as sexual health [30], psychological disorders [31], and have been demonstrated to increase self‐efficacy, readiness, knowledge, and behaviors of ACP [32, 33]. Current ACP interventions for this population focus on advance directive completion or ACP education without first allowing patients to explore their goals for care [34, 35, 36]. This exploration of goals for care, which is accomplished during game play, may further motivate participants to consider their wishes either through conversation with loved ones and to further engage with the healthcare team or create an advance directive after playing the game [14]. Exploring goals and highlighting “why” ACP may help prime individuals to be more ready to accept and engage with more traditional educational tools or interventions such as workbooks or decision aids.

However, to best maximize these results, it is important to ensure that all interventions, including *Hello*, are tailored to the target population, in this case, patients with cancer and their caregivers. As such, the modifications recommended by the study participants reinforce the importance of testing interventions within disease‐specific contexts as the needs of such participants can vary and often require modification. This reaffirms findings from our team's experience testing *Hello* in groups of patients with Alzheimer's disease and related dementias, where special considerations for this population have been identified as well (manuscript in progress).

Our findings should not be generalized because of the small sample consisting solely of spousal dyads facing either breast or genitourinary cancer, and because qualitative research is not intended to be generalized. The reason for this was that we recruited from clinics of clinical partners (who specialized in breast, lung, and genitourinary cancer) in order to prioritize the therapeutic alliance between the patients and their clinicians in the discussion of ACP. Further, no patients with lung cancer enrolled in the study, and this finding needs further exploration. There is also the possibility of selection bias, given that a convenience sampling was used for recruitment, and those with more favorable views of games and ACP may have been more likely to participate. Further, because of the preliminary stage of this work, we did not conduct additional follow‐up of subsequent ACP behavior after playing the game.

Conversely, our study also has several strengths, which include our rigorous methodologic approach to analysis and our heterogeneous sample that includes representation from two cancer types (breast and genitourinary), multiple cancer stages, and individuals at different levels of ACP engagement.

Next steps for this research include broadening our evaluation to other cancer populations, such as cancers like gastrointestinal, head and neck, and hematological cancer, and assessing longitudinal ACP outcomes motivated by the game. Further, repeat acceptability testing of the revised *Hello* game is planned to determine if additional modifications are required prior to a randomized controlled trial comparing the game to other interventions or a control. We further intend to incorporate our findings from our ongoing 75‐site cluster randomized controlled trial testing the game in non‐cancer participants in community settings [37].

In conclusion, we found that *Hello* was well‐received by both patients with cancer and their care partners, demonstrating the potential of this health game to help engage this population in ACP. Participant feedback was used to tailor *Hello* for patients with cancer and their CPs. Future studies will repeat acceptability testing of the revised *Hello* game and will assess ACP outcomes after playing the game. This intervention could be helpful for clinicians looking to encourage patients to have conversations with their family by providing an evidence‐based supportive tool that engages families in substantive conversations.

## Author Contributions

All authors had full access to the data in the study and take responsibility for the integrity of the data and the accuracy of the data analysis. Conceptualization: M.H., M.J., E.V., and L.J.V.S. Methodology: M.H., M.J., E.V., and L.J.V.S. Investigation: K.K., M.B., H.K., and E.V. Formal analysis: K.K., M.H., M.B., E.V., E.W., and L.J.V.S. Resources: M.H., T.G., M.J., and L.J.V.S. Data curation: E.W.; Writing – original draft: K.K., M.B., H.K., and L.J.V.S. Writing – review and editing: K.K., M.H., M.B., W.C., T.G., M.J., H.K., T.M., E.V., E.W., and L.J.V.S. Supervision: L.J.V.S. Project administration: K.K., E.V., and L.J.V.S. Funding acquisition: L.J.V.S. and M.H.

## Ethics Statement

This study was approved and overseen by the Penn State University Institutional Review Board (STUDY00023029). This trial is registered to clinicaltrials.gov (NCT06028152).

## Consent

All research participants provided written consent before participating in any research procedures.

## Conflicts of Interest

The Principal Investigator (LJVS) is an unpaid scientific advisor for Common Practice LLC, which is the company that designed the *Hello* game. There are no other relevant conflicts of interest to report that are related to this work.

## Data Availability

The data that support the findings of this study are available from the corresponding author upon reasonable request.

## Appendix 1 Modifications to *Hello* Game Based on Pilot Study Results

**Table:**

Question	Original game	Revised game
1	Write down any fears you might have about playing this game.	When you first heard about your or your partner's cancer diagnosis, who did you worry about telling?
2	Write down any hopes that you have about playing this game.	When was the last time you had a meaningful conversation with someone and didn't talk about cancer?
3	If you had 3 months to live, what would you give yourself permission to do? Choose one per month.	If you knew you had one good week coming up, what would you plan to do?
4^a^	Name the three‐person committee who should be consulted on any decisions made about whether to continue life‐saving care if you can't communicate? Circle the name of the head of the committee.	Name the three‐person committee who should be consulted on any decisions made about whether to continue life‐saving care if you can't communicate? Circle the name of the head of the committee.
5^a^	In order to provide you with the best care possible, what three non‐medical facts should your doctor know about you?	In order to provide you with the best care possible, what three non‐medical facts should your doctor know about you?
6	Which is more frightening to imagine: suffering the worst physical pain of your life or not getting a chance to say goodbye to your family?	What small thing are you most grateful for these days?
7	What is the last meal you want to eat and who would you like to join you?	If your doctor asked you about your spiritual beliefs, what would you say?
8^a^	Do you want your healthcare team to know about your spiritual or religious beliefs? Why or why not?	What would you like done with your body after you die? (1) embalmed and buried (2) natural burial 3) donated to science (4) cremated and: (5) other:_______________
9^a^	What would you like done with your body after you die? (1) embalmed and buried (2) natural burial (3) donated to science (4) cremated and: (5) other:_______________	Which is more frightening to imagine: suffering the worst physical pain of your life or not getting a chance to say goodbye to your family?
10^a^	If you were unable to communicate and your family was asked to make a decision about a lifesaving treatment that would result in you requiring round‐the‐clock nursing care for the rest of your life, what would you want them to decide? Would your decision be different if you knew that this care was fully covered by your insurance?	When was the last time you needed help from someone else? What made accepting help easier for you? What made it more difficult?
11	If you could control only one thing about the place that you spend your last hours of life, what would it be?	If a healthcare provider asked you to define “quality of life”, what recent day would you tell them about?
12^a^	If you were diagnosed with a terminal disease, who would you turn to for advice?	If only one story is told at your memorial service, who should tell it and why?
13	Write your own epitaph in five words or less (an epitaph is a short statement honoring a person who has died. It can be carved on a tombstone or spoken aloud).	When you look at old photos of yourself, what do you notice first?
14^a^	When you think about care at the end of your life, do you worry more about: not getting enough care, getting overly aggressive care, or other.	If you could control only one thing about the place that you spend your last hours of life, what would it be?
15	What is the first memory that comes to mind when you think about physical pain?	When you first heard about your or your partner's cancer diagnosis, what did you say to yourself, but not to anyone else.
16	If someone wanted to make a donation in your memory after you died, where would you like their donation to go and why?	(A). Outside of the people in this room, who do you turn to for advice? (B). Who do you turn to for comfort?
17^a^	If only one story is told at your memorial service, who should tell it and why?	If you needed help going to the bathroom today, who is the first person you would ask to help you? Who would you never be able to ask?
18^a^	If a healthcare provider asked you to define “quality of life”, what would you say?	When you think about care at the end of your life, do you worry more about: c not getting enough care c getting overly aggressive care c other:________________
19	If you could write a note to the people who will care for you at the end of your life that would be delivered 1 year after your death, what would it say?	When was the last time you got angry?
20^a^	How comfortable are you saying “I don't understand” to your doctor?	If someone wanted to make a donation in your memory after you died, where would you like their donation to go and why?
21	What music do you want to be listening to on your last day alive?	When discussing cancer and treatment, what phrase or idea do you keep going back to?
22^a^	What do you think happens to you after you leave this life?	What music do you want to be listening to on your last day alive?
23	If you needed help going to the bathroom today, who is the first person you would ask to help you? Who would you never be able to ask?	When was the last time you did something that helped you feel more in control?
24^a^	If your doctor believed you had 6 months to live, who would you want them to tell?	If you could write a note to the people who will care for you at the end of your life that would be delivered 1 year after your death, what would it say?
25	If you could pick anyone to sing at your memorial service, who would it be, and what would they sing?	A. What has been surprisingly hard for you? B. What has been surprisingly easy?
26^a^	If you had 1 day to say you were sorry to anyone before you wanted to before you died, who would be first?	If you could pick anyone to sing at your memorial service, who would it be, and what would they sing?
27^a^	I want my doctor to be focused on maximizing: The length of my life, the quality of my life, other.	If you had 1 day to say you were sorry to anyone you wanted to before you died, who would be first?
28^a^	When was the last time you needed help from someone else? What made accepting help more easier for you? What made it more difficult?	Who haven't you talked to in more than 6 months that you would want to talk to before you died?
29^a^	Who haven't you talked to in more than 6 months that you would want to talk to before you died?	What is your earliest memory of attending a funeral or memorial service?
30	What is your earliest memorial of attending a funeral or memorial service?	How do you feel when someone uses the word “hope”?
31^a^	Think about the most important decision you've made in the past 2 years. What did you give up when you made that decision?	Think of someone you've loved who has died. What could you keep in your pocket to remember them by?
32	Think of someone you've loved who has died. What could you keep in your pocket to remember them by?	Of your medical staff and caregivers, who has talked to you in a way that you were most able to hear what they had to say?

a Questions that remained the same from the original to the revised version of *Hello* game.

## References

[1] V. F. Reyna , W. L. Nelson , P. K. Han , and M. P. Pignone , “Decision Making and Cancer,” American Psychologist 70, no. 2 (2015): 105–118, 10.1037/a0036834. 25730718 PMC4347999

[2] K. E. Knutzen , O. A. Sacks , O. C. Brody‐Bizar , et al., “Actual and Missed Opportunities for End‐Of‐Life Care Discussions With Oncology Patients,” JAMA Network Open 4, no. 6 (2021): e2113193, 10.1001/jamanetworkopen.2021.13193. 34110395 PMC8193430

[3] R. L. Sudore , H. D. Lum , J. J. You , et al., “Defining Advance Care Planning for Adults: A Consensus Definition From a Multidisciplinary Delphi Panel ‐ PubMed,” Journal of Pain and Symptom Management 53, no. 5 (2017): 821–832, 10.1016/j.jpainsymman.2016.12.331. 28062339 PMC5728651

[4] Medicine Io and Medicine Io , Dying in America: Improving Quality and Honoring Individual Preferences Near the End of Life (National Academies Press, 2014), 10.17226/187482014/09/17. 25927121

[5] C. C. Earle , B. A. Neville , M. B. Landrum , J. Z. Ayanian , S. D. Block , and J. C. Weeks , “Trends in the Aggressiveness of Cancer Care Near the End of Life,” Journal of Clinical Oncology 22, no. 2 (2004): 315–321, 10.1200/JCO.2004.08.136. 14722041

[6] B. Wendlandt , A. Ceppe , B. N. Gaynes , et al., “Posttraumatic Stress Disorder Symptom Clusters in Surrogate Decision Makers of Patients Experiencing Chronic Critical Illness,” Critical Care Explorations 4, no. 3 (2022): e0647, 10.1097/CCE.0000000000000647. 35261980 PMC8893298

[7] R. L. Hickman and M. D. Pinto , “Advance Directives Lessen the Decisional Burden of Surrogate Decision‐Making for the Chronically Critically Ill ‐ PubMed,” Journal of Clinical Nursing 23, no. 5‐6 (2014): 756–765, 10.1111/jocn.12427. 24330417 PMC5573593

[8] E. Azoulay , F. Pochard , N. Kentish‐Barnes , et al., “Risk of Post‐Traumatic Stress Symptoms in Family Members of Intensive Care Unit Patients ‐ PubMed,” American Journal of Respiratory and Critical Care Medicine 171, no. 9 (2005): 987–994, 10.1164/rccm.200409-1295OC. 15665319

[9] J. M. Peppercorn , T. J. Smith , P. R. Helft , et al., “American Society of Clinical Oncology Statement: Toward Individualized Care for Patients With Advanced Cancer,” Journal of Clinical Oncology 29, no. 6 (2011): 755–760, 10.1200/JCO.2010.33.1744. 21263086

[10] J. C. McDonald , J. M. du Manoir , N. Kevork , L. W. Le , and C. Zimmerman , “Advance Directives in Patients With Advanced Cancer Receiving Active Treatment: Attitudes, Prevalence, and Barriers ‐ PubMed,” Supportive Care in Cancer: Official Journal of the Multinational Association of Supportive Care in Cancer 25, no. 2 (2017): 523–531, 10.1007/s00520-016-3433-6. 27718068

[11] C. Bernard , A. Tan , M. Slaven , D. Elston , D. K. Heyland , and M. Howard , “Exploring Patient‐Reported Barriers to Advance Care Planning in Family Practice,” BMC Family Practice 21, no. 1 (2020): 94, 10.1186/s12875-020-01167-0. 32450812 PMC7249389

[12] E. Ko , A. J. Keeney , D. Higgins , N. Gonzalez , and H. Palomino , “Rural Hispanic/Latino Cancer Patients' Perspectives on Facilitators, Barriers, and Suggestions for Advance Care Planning: A Qualitative Study,” Palliative & Supportive Care Cambridge Core. Palliative & Supportive Care 20, no. 4 (2022): 535–541, 10.1017/S1478951521001498. 35876451

[13] L. J. Van Scoy , J. M. Reading , A. M. Scott , M. J. Green , and B. H. Levi , “Conversation Game Effectively Engages Groups of Individuals in Discussions About Death and Dying,” Journal of Palliative Medicine 19, no. 6 (2016): 661–667, 10.1089/jpm.2015.0390. 27022862

[14] L. J. Van Scoy , J. M. Reading , A. M. Scott , C. Chuang , B. H. Levi , and M. J. Green , “Exploring the Topics Discussed During a Conversation Card Game About Death and Dying: A Content Analysis,” Journal of Pain and Symptom Management 52, no. 5 (2016): 655–662, 10.1016/j.jpainsymman.2016.03.021. 27650010

[15] L. J. Van Scoy , M. J. Green , J. M. Reading , A. M. Scott , C. H. Chuang , and B. H. Levi , “Can Playing an End‐Of‐Life Conversation Game Motivate People to Engage in Advance Care Planning?,” American Journal of Hospice & Palliative Care 34, no. 8 (2017): 754–761, 10.1177/1049909116656353. 27406696 PMC6055477

[16] L. J. Van Scoy , A. M. Scott , J. M. Reading , et al., “From Theory to Practice: Measuring End‐Of‐Life Communication Quality Using Multiple Goals Theory,” Patient Education and Counseling 100, no. 5 (2017): 909–918, 10.1016/j.pec.2016.12.010. 28011081

[17] L. J. Van Scoy , J. M. Reading , M. Hopkins , et al., “Community Game Day: Using an End‐Of‐Life Conversation Game to Encourage Advance Care Planning,” Journal of Pain and Symptom Management 54, no. 5 (2017): 680–691, 10.1016/j.jpainsymman.2017.07.034. 28743662

[18] K. Radhakrishnan , L. J. Van Scoy , R. Jillapalli , S. Saxena , and M. T. Kim , “Community‐Based Game Intervention to Improve South Asian Indian Americans' Engagement With Advanced Care Planning,” Ethnicity & Health 24, no. 6 (2019): 705–723, 10.1080/13557858.2017.1357068. 28748743

[19] L. J. Van Scoy , E. Watson‐Martin , T. A. Bohr , et al., “End‐Of‐Life Conversation Game Increases Confidence for Having End‐Of‐Life Conversations for Chaplains‐In‐Training,” American Journal of Hospice & Palliative Medicine 35, no. 4 (2017): 592–600, 10.1177/1049909117723619. 28782376

[20] L. J. Van Scoy , B. H. Levi , P. Witt , et al., “End‐Of‐Life Conversation Game, Advance Care Planning Behavior, and Perspectives Among African American Individuals,” JAMA Network Open 3, no. 5 (2020): e204315, 10.1001/jamanetworkopen.2020.4315. 32383747 PMC7210487

[21] L. J. Van Scoy , M. J. Green , and R. Volpe , “Evaluating an Advance Care Planning Curriculum: A Lecture, a Game, a Patient, and an Essay,” Medical Science Educator 29 (2019): 453–462. 34457502 10.1007/s40670-019-00713-5 PMC8368620

[22] R. L. Sudore , D. K. Heyland , D. E. Barnes , et al., “Measuring Advance Care Planning: Optimizing the Advance Care Planning Engagement Survey,” Journal of Pain and Symptom Management 53, no. 4 (2017): 669–681, 10.1016/j.jpainsymman.2016.10.367. 28042072 PMC5730058

[23] R. L. Sudore , A. L. Stewart , S. J. Knight , et al., “Development and Validation of a Questionnaire to Detect Behavior Change in Multiple Advance Care Planning Behaviors,” PLoS One 8, no. 9 (2013): e72465, 10.1371/journal.pone.0072465. 24039772 PMC3764010

[24] L. J. Van Scoy , A. G. Day , M. Howard , R. Sudore , and D. K. Heyland , “Adaptation and Preliminary Validation of the Advance Care Planning Engagement Survey for Surrogate Decision Makers,” Journal of Pain and Symptom Management 57, no. 5 (2019): 980–988.e9, 10.1016/j.jpainsymman.2019.01.008. 30684633 PMC6857702

[25] M. L. Hecht , “Measures of Communication Satisfaction,” Human Communication Research 4, no. 4 (1978): 350–368, 10.1111/j.1468-2958.1978.tb00721.x.

[26] M. L. Hecht , “The Conceptualization and Measurement of Interpersonal Communication Satisfaction,” Human Communication Research 4, no. 3 (1978): 253–264, 10.1111/j.1468-2958.1978.tb00614.x.

[27] MAXQDA (VERBI Software, 2024), http://maxqda.com.

[28] B. A. Primack , M. V. Carroll , M. McNamara , et al., “Role of Video Games in Improving Health‐Related Outcomes: A Systematic Review,” American Journal of Preventive Medicine 42, no. 6 (2012): 630–638, 10.1016/j.amepre.2012.02.023. 22608382 PMC3391574

[29] E. Rahmani and S. A. Boren , “Videogames and Health Improvement: A Literature Review of Randomized Controlled Trials,” Games for Health Journal 1, no. 5 (2012): 331–341, 10.1089/g4h.2012.0031. 26191999

[30] A. DeSmet , R. Shegog , D. Van Ryckeghem , G. Crombez , and I. De Bourdeaudhuij , “A Systematic Review and Meta‐Analysis of Interventions for Sexual Health Promotion Involving Serious Digital Games,” Games for Health Journal 4, no. 2 (2015): 78–90, 10.1089/g4h.2014.0110. 26181801

[31] K. N. Dunlap , “Integration of Game Design and Theory Into Group Psychotherapy With Veterans With Severe/Chronic Mental Illness,” Games for Health Journal 2, no. 2 (2013): 109–112, 10.1089/g4h.2013.0003. 26192129

[32] L. Liu , Y. Y. Zhao , C. Yang , and H. Y. Chan , “Gamification for Promoting Advance Care Planning: A Mixed‐Method Systematic Review and Meta‐Analysis,” Palliative Medicine 35, no. 6 (2021): 1005–1019, 10.1177/02692163211005343. 33775174

[33] L. Liu , H. Y. Chan , T. C. Ho , et al., “A Serious Game for Engaging Older Adults in End‐Of‐Life Care Discussion: A Mixed Method Study,” Patient Education and Counseling 113 (2023): 107787, 10.1016/j.pec.2023.107787. 37148841

[34] M. A. MacKenzie , E. Smith‐Howell , P. A. Bomba , and S. H. Meghani , “Respecting Choices and Related Models of Advance Care Planning: A Systematic Review of Published Evidence,” American Journal of Hospice & Palliative Medicine 35, no. 6 (2017): 897–907, 10.1177/1049909117745789. 29254357 PMC6580846

[35] M. I. Patel , K. Kapphahn , M. Dewland , et al., “Effect of a Community Health Worker Intervention on Acute Care Use, Advance Care Planning, and Patient‐Reported Outcomes Among Adults With Advanced Stages of Cancer: A Randomized Clinical Trial,” JAMA Oncology 8, no. 8 (2022): 1139–1148, 10.1001/jamaoncol.2022.1997. 35771552 PMC9247857

[36] N. Michael , C. O'Callaghan , A. Baird , et al., “A Mixed Method Feasibility Study of a Patient‐ and Family‐Centred Advance Care Planning Intervention for Cancer Patients,” BMC Palliative Care 14, no. 1 (2015): 27, 10.1186/s12904-015-0023-1. 25981642 PMC4456060

[37] L. J. Van Scoy , B. H. Levi , C. Bramble , et al., “Comparing Two Advance Care Planning Conversation Activities to Motivate Advance Directive Completion in Underserved Communities Across the USA: The Project Talk Trial Study Protocol for a Cluster, Randomized Controlled Trial,” Trials 23, no. 1 (2022): 829, 10.1186/s13063-022-06746-3. 36180899 PMC9523194

